# Supplementary material for: Assessment of distant-site rescue elements for CRISPR toxin-antidote gene drives
Source: Front Bioeng Biotechnol. 2023 Feb 13;11:1138702. doi: 10.3389/fbioe.2023.1138702 (PMC9968759; doi:10.3389/fbioe.2023.1138702)
Supplement: Supplementary file 1 [file DataSheet1.DOCX]

Supplementary Material

Assessment of distant-site rescue elements for CRISPR toxin-antidote gene drives

Jingheng Chen, Xuejiao Xu, Jackson Champer*

*****Correspondance: jchamper@pku.edu.cn

**Plasmid key and injections**

| **Injection** | **Fly Line** | **Donor** | **gRNA Helper** |
| --- | --- | --- | --- |
| polyubiquitin-EGFP | *w^1118^* | STSacG | BHDacg1 |
| RpL35A rescue-only | *w^1118^* | AHDr35s | BHDadg1 |
| TADE Suppression Drive | *w^1118^* | TADESygrU2 | TTTygU4 |
| 2-gRNA TADE | AHDr35s | TADEr2 | TTTgU2s |
| 4-gRNA TADE | STSacG | TADEr4 | TTTgU1 |
| TARE | STSacG | TAREh4 | TTTgU1 |
| TARE reversed rescue | STSacG | TAREh4v2 | TTTgU1 |

For all injections, TTChsp70c9 was used as a source of Cas9.

***Rpl35A* target site analysis primers**

RpL35ALeft_S_F: GCATGCAAATGATCGAAACCCT

RpL35ARight_S2_R: CGTTTCCATCGTCTTCATCTGC

**STSacG insertion site check primers**

AutoCLeft_S2_F: AGATTGGCCACCACATCCATC

EGFPaLeft_S_R: GCTTGTTTATTTGCTTAGCTTTCGC

**AHDr35s insertion site check primers**

AutoDLeft_S2_F: TTTGATCTGATTGCGACGCGT

RpL35ARight_S_F: CGCATCCGCATCGTTAGTTCA

**TADESygrU2 insertion site check primers**

U6term_S_F: CATCTGACGTGTGTTTATTTAGAC

YGRight_S2_R: TAATGAGACCCAGTAACGACA

**TADEr2 insertion site check primers**

U6term_S_F: CATCTGACGTGTGTTTATTTAGAC

AutoDRight_S2_R: GCAATGGTAATGACTCACAGT

**TADEr4, TAREh4, and TAREh4v2 insertion site check primers**

U6term_S_F: CATCTGACGTGTGTTTATTTAGAC

AutoCRight_S_R: TACACCTCACACTACTCGGGC
